# A robust blood gene expression-based prognostic model for castration-resistant prostate cancer

**DOI:** 10.1186/s12916-015-0442-0

**Published:** 2015-08-21

**Authors:** Li Wang, Yixuan Gong, Uma Chippada-Venkata, Matthias Michael Heck, Margitta Retz, Roman Nawroth, Matthew Galsky, Che-Kai Tsao, Eric Schadt, Johann de Bono, David Olmos, Jun Zhu, William K. Oh

**Affiliations:** Icahn Institute for Genomics and Multiscale Biology, New York, NY 10029 USA; Department of Genetics and Genomic Sciences, New York, USA; The Tisch Cancer Institute, Icahn School of Medicine at Mount Sinai, New York, NY 10029 USA; Department of Urology, Klinikum rechts der Isar, Technische Universität München, Munich, Germany; Institute for Cancer Research, Royal Marsden Hospital, Sutton, Surrey UK; Prostate Cancer clinical research Unit, Spanish National Cancer Research Centre (CNIO), Madrid, Spain; Medical Oncology Deparment, CNIO-IBIMA Genitourinary Cancer Clinical Research Unit, hospitales Universitarios Virgen de la Victoria y Regional de Málaga, Málaga, Spain

## Abstract

**Background:**

Castration-resistant prostate cancer (CRPC) is associated with wide variations in survival. Recent studies of whole blood mRNA expression-based biomarkers strongly predicted survival but the genes used in these biomarker models were non-overlapping and their relationship was unknown. We developed a biomarker model for CRPC that is robust, but also captures underlying biological processes that drive prostate cancer lethality.

**Methods:**

Using three independent cohorts of CRPC patients, we developed an integrative genomic approach for understanding the biological processes underlying genes associated with cancer progression, constructed a novel four-gene model that captured these changes, and compared the performance of the new model with existing gene models and other clinical parameters.

**Results:**

Our analysis revealed striking patterns of myeloid- and lymphoid-specific distribution of genes that were differentially expressed in whole blood mRNA profiles: up-regulated genes in patients with worse survival were overexpressed in myeloid cells, whereas down-regulated genes were noted in lymphocytes. A resulting novel four-gene model showed significant prognostic power independent of known clinical predictors in two independent datasets totaling 90 patients with CRPC, and was superior to the two existing gene models.

**Conclusions:**

Whole blood mRNA profiling provides clinically relevant information in patients with CRPC. Integrative genomic analysis revealed patterns of differential mRNA expression with changes in gene expression in immune cell components which robustly predicted the survival of CRPC patients. The next step would be validation in a cohort of suitable size to quantify the prognostic improvement by the gene score upon the standard set of clinical parameters.

**Electronic supplementary material:**

The online version of this article (doi:10.1186/s12916-015-0442-0) contains supplementary material, which is available to authorized users.

## Background

Prostate cancer is an extremely heterogeneous disease [1]. For patients with castration-resistant prostate cancer (CRPC), overall survival can range widely from months to years. Accurate prediction of survival is crucial for clinical management and for patient stratification into clinical trials. Unfortunately, monitoring genetic alterations in metastatic prostate cancer has been inhibited by the difficulty in obtaining serial metastatic biopsies, since these are not routinely needed for clinical management. Blood-based biomarker assays are minimally invasive and can be easily implemented in clinical practice. As such, diagnostic and prognostic models built on peripheral blood gene expression have been reported for various types of cancers [2–9]. Two recently published studies from our respective groups [10, 11] suggested that the RNA transcript levels of specific gene sets in whole blood samples were significantly associated with overall survival in patients with CRPC. However, the lists of genes identified by the two studies were completely non-overlapping and questions remained regarding the underlying pathogenic processes reflected by the two distinct signatures.

Such lack of consistency is not uncommon in genome-wide biomarker discovery studies given the large pool of candidate genes with complex correlation structures, relatively small sample sizes, the noisy nature of high-throughput technologies, and cross-platform variables. Specifically, a six-gene signature reported by Ross et al. [11] was derived from qRT-PCR profiling and modeling of 168 pre-selected genes associated with inflammation, immune response, angiogenesis, apoptosis, tumor suppression, cell cycle, DNA repair, and tumor progression using whole-blood RNA samples from CRPC patients. Gene expression changes in patients with increased mortality was associated with down-regulation of cellular and humoral immunity and monocyte differentiation towards the production of tissue macrophages. A second signature developed by Olmos et al. [10] was constructed by selecting top ranking differentially-expressed genes from microarray whole blood RNA profiling data comparing a group of CRPC patients showing worse survival. This resulting gene signature associated a poor prognosis to increased CD71(+) erythroid progenitor cells. While both models strongly predicted prognosis, the very different gene signatures suggested different underlying immunological drivers.

Computational techniques can improve the results of genome-wide biomarker discovery studies, although each has its own shortcomings. For instance, meta-analysis identifies robust biomarkers that correlate with the phenotype of interest across multiple datasets [12]. However, multiple datasets must be available with similar experimental designs. Advanced machine learning techniques, such as ElasticNet [13], can construct predictive models from genomic data, but these models are overly reliant on the training dataset; the resulting algorithms cannot distinguish genuine from random correlations with phenotype. Furthermore, there is often no clear molecular mechanism underlying these biomarker models. As a result, it is difficult to develop biological interpretations of the generated models.

To overcome these issues, we developed a novel computational strategy that builds robust prognostic models by selecting genes within stable co-expression modules. This method integrates independent mRNA expression datasets that come from different experimental designs, and derives stable co-expression modules among candidate signature genes. Representative genes are then selected from each stable co-expression module to build a predictive model. This method thus generates gene expression models which, together with underlying biological pathways, facilitate hypothesis formation. We applied this novel strategy to reanalyze the Olmos et al. [10] dataset and generated a superior four-gene prognostic model. The new model was then validated in two independent CRPC cohorts.

## Methods

### Workflow of a co-expression module-based integrative approach to build robust prognostic models

#### Step 1. Create a list of candidate prognostic genes

The Olmos dataset [10] was downloaded from GEO (GSE37199) and the non-CRPC samples were removed from the dataset. A list of candidate prognostic genes was created by applying differential expression analysis to the two groups of CRPC patients with different survival outcomes in Olmos dataset. We used the R package LIMMA [14] and identified 2,209 candidate prognostic genes at a false discovery rate of <0.05 [15].

#### Step 2. Identify stable co-expression modules among candidate prognostic genes

We extracted whole blood gene expression profiles of 437 males from the Iceland Family Blood (IFB) study [16] and 99 male samples from the Genotype-Tissue Expression (GTEx) study [17]. Based on each of the two datasets, we identified co-expression modules among the up-regulated and down-regulated candidate genes from step 1, separately using the R package WGCNA [18]. We then compared modules derived from the two datasets and ranked the overlap between modules according to their significance (Fisher’s exact test). We noted significant overlap (*P* value of Fisher’s exact test <0.01) of stable co-expression modules. If the list of up-regulated stable co-expression modules was not of the same length as that of the down-regulated ones, we discarded the bottom ranking stable co-expression modules from the longer list to make them the same length.

#### Step 3. Identify functional cores of stable co-expression modules

We carried out gene set enrichment analysis for each stable co-expression module from step 2 using two types of gene sets. The first gene set was the canonical pathway downloaded from the MsigDB database [19]. The second set consisted of genes overexpressed in specific types of hematopoietic cells, obtained from the HematoAtlas study [20]. The functional core of each module was defined as the intersection between the module and its most significantly enriched canonical pathway (*P* value of Fisher’s exact test <1×10^−4^, corresponding to a family wise error rate of 0.1 after Bonferroni correction). In case there was no significantly enriched canonical pathway for the module (the first type of gene set), we used the intersection between the module and its most significantly enriched gene set of cell type-specific overexpression (the second type of gene set).

#### Step 4. Select representative genes for each co-expression module

From the functional core of each stable co-expression module (step 3), a representative gene was selected as the most differentially expressed between good and poor prognosis groups in step 1. To avoid selecting genes with very low expression levels, we also required the expression level of the representative gene to be higher than half of genes in the genome. We thus obtained two lists of representative genes from up-regulated and down-regulated modules, respectively, which were ordered according to their corresponding modules, i.e. *P* value of the overlapping significance (step 2).

#### Step 5. Train and cross-validate prognostic models

We then built gene models based on the representative genes (step 4), using the Olmos dataset as the training dataset and the naïve Bayesian classifier (R package e1071) as the learning algorithm. The pre-assumption of features independent of the Bayesian classifier was largely satisfied since the representative genes were chosen from modules with distinct expression profiles. We used leave-one-out cross-validation to determine the optimal number of genes included in the model (Additional file 1).

### Validation sets I and II

The first validation dataset (I) consisted of 25 CRPC patients recruited from Mount Sinai Medical Center in New York. Whole-blood RNA was extracted using the PAXgene RNA extraction kit. After proper RNA quality control, the samples were sent for RNA-seq at the Genomic Core Facility at Mount Sinai. Illumina HiSeq 2500 was used for RNA-seq with 100 nt single read and poly(A) enriched library. The TopHat software was used to generate fragments per kilobase of exon per million fragments mapped (FPKM) values for each gene. We applied a gene-wise standardization strategy [21, 22] to adjust the platform difference between the training and validation datasets. More specifically, for each gene in the validation dataset, we linearly transformed the log2 FPKM value to make its median and median absolute deviation the same as that of the training dataset. We then calculated the four-gene score based on the gene expression after transformation. Similarly, to calculate Ross six-gene score in the validation dataset, we scaled the log2 FPKM values according to the gene distribution in the Ross training dataset [11]. Since the original data (by qRT-PCR using a custom Taqman array) to optimize the parameters and the cutoff value of the Olmos nine-gene score were no longer available, such transformation was not applicable to this score.

To get four-gene PCR measurements for validation set I, first-strand cDNA was synthesized from oligo-dT primed RNA templates using SuperScript® III First-Strand Synthesis System for RT-PCR (Life Technologies). Expression levels of individual genes in the four-gene signature were determined on the ViiA7 qPCR instrument using custom-made Taqman Array Cards (Life Technologies) with the Taqman Universal qPCR master mix. The delta Ct value was normalized using 18S RNA as endogenous control. To adjust the platform difference, we did a similar transformation of delta Ct value according to its distribution in the training dataset.

The second validation dataset (II) consisted of 66 CRPC patients recruited from the Urology Clinic at the University of Technology in Munich, Germany. Whole blood samples were collected in PAXgene™ Blood RNA tubes. The four-gene qPCR measurements were obtained as described for the first validation set.

### Ethical considerations

The first validation dataset (I) consisted of 25 CRPC patients recruited from Mount Sinai Medical Center in New York. The PPHS (Program for the Protection of Human Subjects) at Mount Sinai Medical Center approved the study (protocol #10-1180; PI: W.Oh) to allow blood collection. All patients provided written informed consent to allow linking of clinical data and serum specimens for research purposes through participation in this specimen-banking protocol.

The second validation dataset (II) consisted of 66 CRPC patients recruited from the Urology Clinic at the University of Technology in Munich, Germany. The study was approved by the Ethics Committee (ethikkommisson, fakultät für Medizin) (project # 313/13; PI: M. Heck) to allow blood collection and all patients provided written informed consent.

The IFB dataset was downloaded from GEO database with accession number GSE7965. The Olmos dataset was downloaded from GEO database with accession number GSE37199. The GTEx dataset was downloaded from dbGap database with study accession phs000424.v5.p1. These three datasets are publicly available. Further consent for using these datasets was not required.

## Results

### Candidate prognostic genes formed stable co-expression modules

In this study, we reanalyzed the dataset of Olmos et al. [10], one of two recently published studies of blood gene expression prognostic biomarkers in CRPC patients [10, 11]. There were a total of 63 CRPC patients in the Olmos dataset. In the original report, an unsupervised classification method was first used to identify a subgroup consisting of 14 CRPC patients with significantly worse survival outcomes. A nine-gene signature (Olmos nine-gene score) was then derived to separate the 14 CRPC patients from the others. These 14 patients were thus referred to as the ‘high-risk group’ and the others as the ‘low-risk group’ in the current study.

**Fig. 1:**
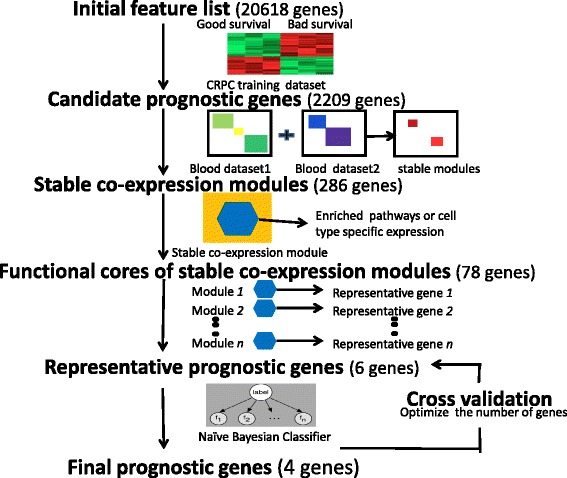
Flowchart of building robust prognostic models from stable co-expression modules

Instead of selecting the best fitting models using candidate prognostic genes which might result in overfitting, we aimed to understand what biological processes were associated with prostate cancer progression in order to represent these biological processes in a prognostic model. As described in Methods, Figure 1 outlines the five-step procedure for our module-based integrative analysis strategy. Our approach begins with a standard two-group differential expression analysis. By comparing expression profiles of high and low-risk patients, we created a candidate prognostic gene pool, which consisted of 1,408 significantly up-regulated and 801 significantly down-regulated genes in the high-risk group (false detection rate <0.05). The nine genes in the Olmos score ranked at the top of our candidate gene list as expected, since they were derived from the same dataset. In contrast, only two of the six signature genes from the Ross study [11] (Ross six-gene score) were in this differential gene list, and both ranked low (*CDKN1A* ranked 1154th and *C1QA* ranked 1243rd in the up-regulated gene list), while the other four genes had a false discovery rate of >0.05.

**Fig. 2:**
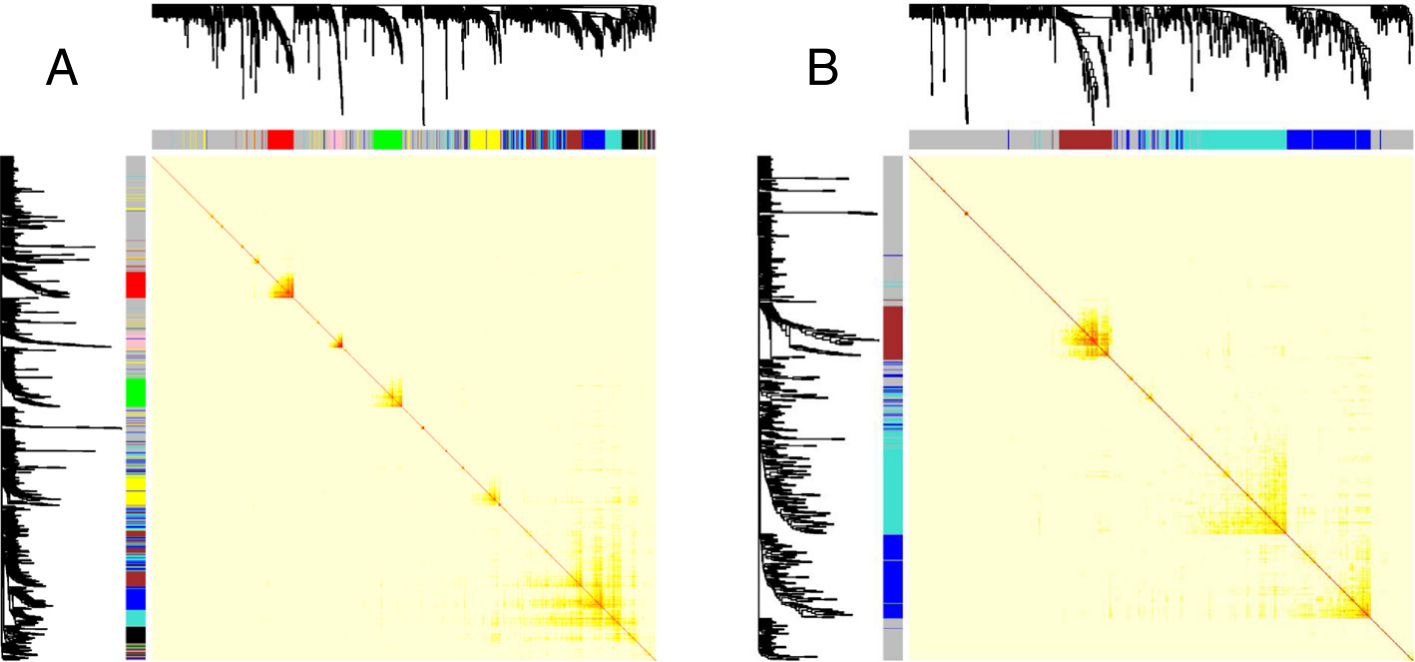
Co-expression networks among genes up-regulated in high-risk CRPC patients (**a**) and genes down-regulated in high-risk CRPC patients (**b**) are constructed from whole blood mRNA profiling of 437 male samples in the IFB dataset. Light color represents low overlap and progressively darker red color represents higher overlap. The gene dendrogram and module assignment are shown along the left side and the top. Each color represents one module, and a grey color represents genes that are not assigned to any modules

**Fig. 3:**
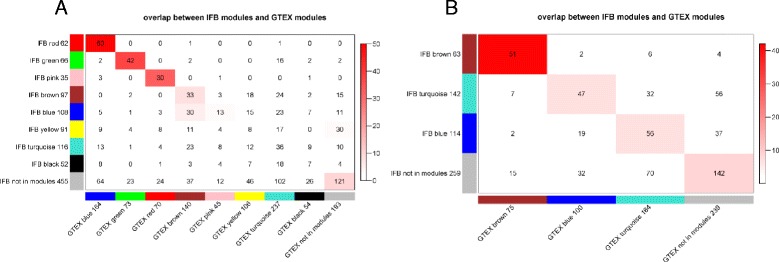
Overlap between IFB modules and GTEx modules for up-regulated genes (**a**) and down-regulated genes (**b**). Each row of the table corresponds to one IFB module, and each column corresponds to one GTEx module. Numbers in the table indicate gene counts in the intersection of the corresponding modules. Coloring of the table encodes –log(p), with *P* being the Fisher’s exact test *P* value for the overlap of the two modules. The modules are ordered according to its maximum –log(p) with other modules. ‘Grey module’ consists of genes that are not assigned to any modules

To understand the biological processes involved among these candidate prognosis genes, we applied co-expression network analysis [16, 23–25] and identified stable co-expression modules across multiple blood gene expression data sets. Stable co-expression modules were defined as those whose expression profiles are correlated consistently under various conditions, and thus less likely to be dataset-specific or due to artifact. We leveraged two large human whole blood gene expression datasets: the IFB dataset [16], consisting of 437 males, and the GTEx dataset [17], consisting of 99 males. Of note, only male samples were used. First, from each dataset, we built co-expression networks and identified co-expression modules for the 1,408 up-regulated and 801 down-regulated genes, respectively. Figure 2 shows the co-expression patterns based on the IFB dataset (the co-expression patterns based on the GTEx dataset are shown in Additional file 1: Figure S1). There were clear modular structures in all four co-expression networks (Fig. 2 and Additional file 1: Figure S1). Modules derived from the two datasets overlapped significantly (Fig. 3). In this study, we refer to co-expression modules as stable if the corresponding modules in the two datasets overlapped significantly (*P* value of Fisher’s exact test <0.01). Using such criteria, we obtained four stable co-expression modules for genes up-regulated in the high-risk group and three stable co-expression modules for genes down-regulated in the same group. It has been shown that classifiers constructed according to relative expression levels of pairs of genes are more robust than individual genes [26, 27]. Thus, we selected the same number of up-regulated and down-regulated modules to create a paired analysis so that resulting scores were less likely affected by normalization procedures [26, 27].

The six stable co-expression modules consisted of 286 genes: three up-regulated modules (referred to hereafter as “up_module_1”, “up_module_2”, and “up_module_3”) and three down-regulated modules (referred to hereafter as “down_module_1”, “down_module_2”, and “down_module_3”) corresponding to the top three cells in the diagonal in Figs. 3a and b, respectively. We annotated the stable co-expression modules against canonical pathways using gene set enrichment analysis (results shown in Additional file 1: Table S1). The up_module_1 was significantly enriched for genes involved in cell cycle (*P* = 8×10^−27^) and the up_module_2 was significantly enriched for genes involved in response to elevated cytosolic Ca^2+^ (*P* = 7×10^−6^). In contrast, the down_module_1 and down_module_3 were enriched for genes involved in the B-cell receptor signaling pathway (*P* = 1×10^−8^) and TCR signaling in naïve CD8^+^ T cells (*P* = 1×10^−5^), respectively. The results suggest that multiple biological processes account for differences in prognosis among CRPC patients.

### Genes in up- and down-regulated modules were overexpressed in myeloid cells and lymphocytes, respectively

Since a whole blood mRNA expression profile reflects genes pooled from a mixture of hematopoietic cells from different lineages, we dissected potential sources of the observed changes in expression level. In addition to comparing the stable co-expression modules with the canonical pathways, we compared them with genes overexpressed in different types of hematopoietic cells (results listed in Additional file 1: Table S2). Both enrichment analyses indicated that different co-expression modules were likely driven by biological process changes in different types of hematopoietic cells. For instance, the “down_module_1” was significantly enriched for both the “B cell receptor signaling pathways” (*P* = 1×10^−8^, Additional file 1: Table S1) and “B cell overexpressed gene set” (*P* = 8×10^−25^, Additional file 1: Table S2); the “down_module_3” was enriched for both the “TCR pathway” (*P* = 1×10^−5^, Additional file 1: Table S1) and “T cell overexpressed genes” (*P* = 5×10^−9^, Additional file 1: Table S2). Similarly, the “up_module_2” was enriched for “platelet activation signaling” (*P* = 4×10^−5^, Additional file 1: Table S1) and “erythroid cell overexpressed genes” (*P* = 9×10^−7^ Additional file 1: Table S2).

**Fig. 4:**
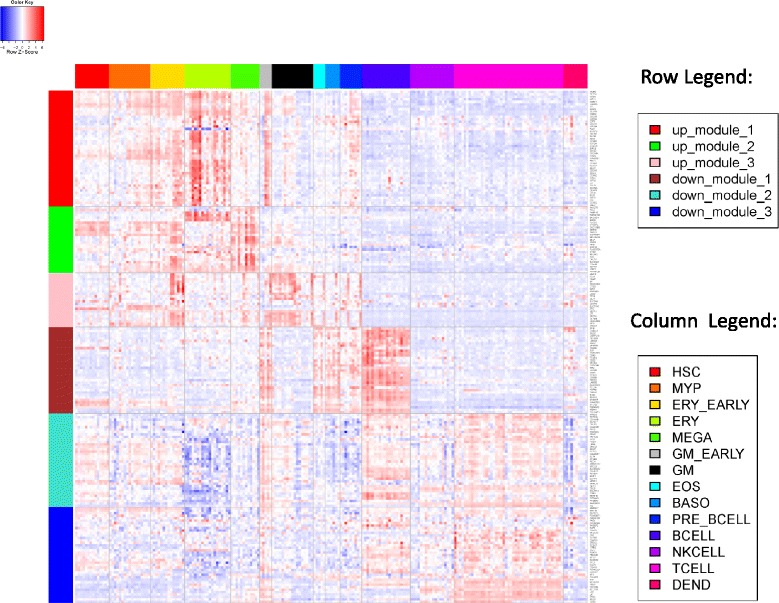
Heatmap of gene expression across different types of blood cell lines for stable co-expression modules. Rows represent genes which are within the stable co-expression modules (row legend). Columns represent blood cell lines which are grouped according to the lineage (column legend). HSC, Hematopoietic stem cell; MYP, Myeloid progenitor; ERY, Erythroid cell; MEGA, Megakaryocyte; GM, Granulocyte/monocyte; EOS, Eosinophil, BASO, Basophil; DEND, Dendritic cell

In fact, when comparing the expression levels of genes in these modules across a panel of hematopoietic cells of different lineages (Fig. 4), we identified a clear pattern of cell type-specific overexpression for each stable co-expression module. Genes in the three up-regulated modules were overexpressed in different lineages of myeloid cells, e.g. erythroid cells, megakaryocytes, and granulocytes/monocytes. Genes in the three down-regulated modules were overexpressed in lymphocytes, e.g. B cells and T cells. Such a pattern was not limited to stable co-expression modules (Additional file 1: Table S3), but cell type-specific overexpression was higher in these modules compared to all genes considered together (enrichment score in Additional file 1: Table S2 and Table S3). In summary, high-risk CRPC patients demonstrated increased expression of myeloid-overexpressed genes and decreased expression of lymphocyte-overexpressed genes.

To best represent the biological processes underlying differing prognosis in CRPC patients, we selected a functional core consisting of genes involved in the top enriched functional gene set for each stable co-expression module. There were a total of 78 genes in the cores and their cell type-specific overexpression patterns are shown in Additional file 1: Figure S2.

### Genes in the two published gene models were overexpressed in different hematopoietic cells

**Fig. 5:**
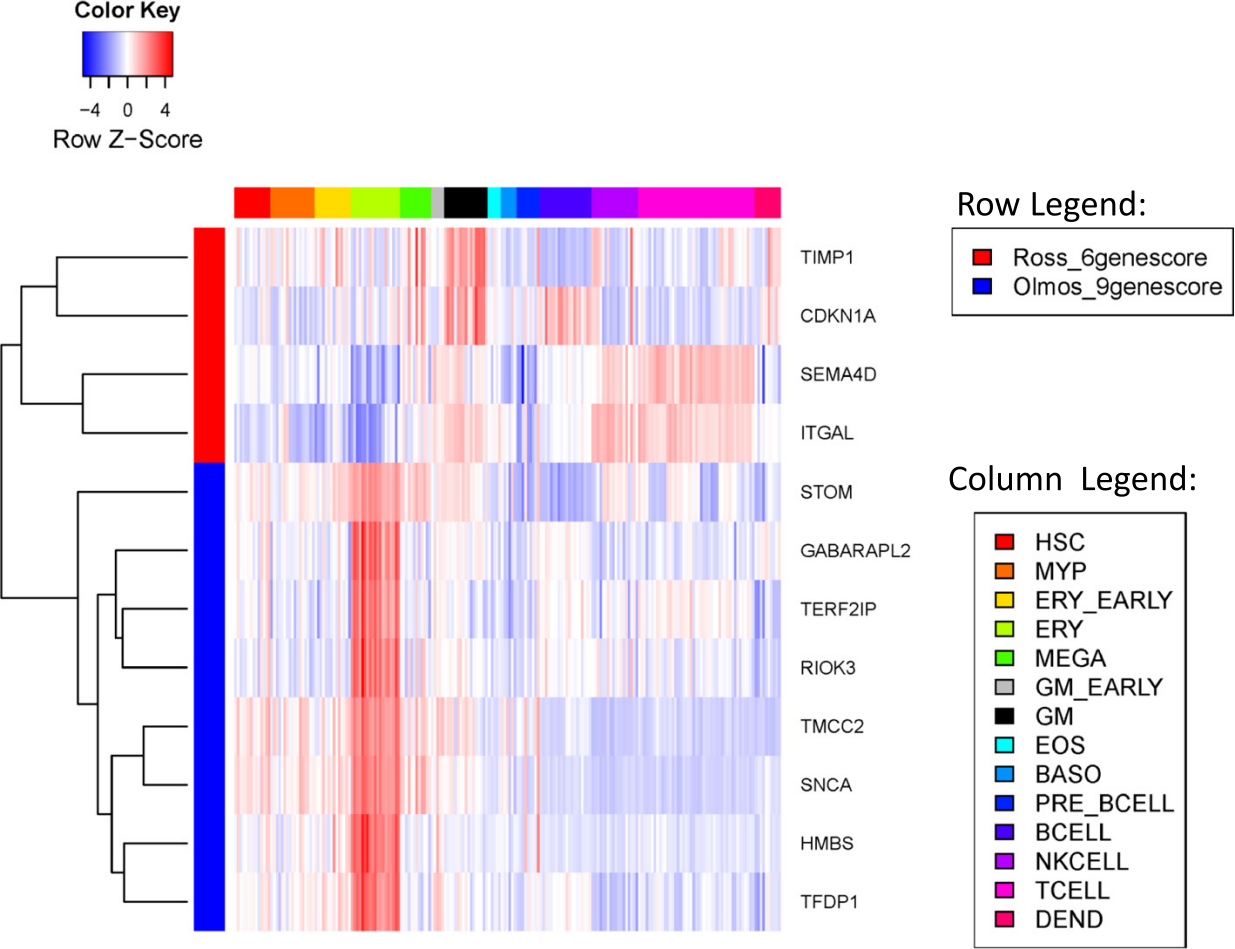
Heatmap of gene expression across different blood cell lines for genes in the two published prognostic models. Rows are genes from different prognostic models (row legend) and columns are cell lines of different lineages (column legend, same as in Fig. 4). Only genes with available cell line expression profiles are shown here

We conducted a similar analysis of cell type-specific overexpression to understand the interrelationships among genes used in the two published prognostic models. Figure 5 shows the expression profiles of genes used in Olmos nine-gene score and Ross six-gene score across different hematopoietic cells. Genes used in Olmos nine-gene score (blue) and those used in Ross six-gene score (red) were overexpressed in different cell types (Fig. 5). Specifically, all genes in the Olmos nine-gene score were overexpressed in erythroid cells. For genes in the Ross six-gene score, two genes (*SEMA4D* and *ITGAL*) were overexpressed in T cells, while the other two (*TIMP1* and *CDKN1A*) were overexpressed in the granulocyte-monocyte and megakaryocyte lines. In fact, in the linear formula used to calculate the six-gene score, the signs for *SEMA4D* and *ITGAL* are opposite that of *TIMP1* and *CDKN1A*, consistent with our observation that myeloid overexpressed genes were up-regulated and the lymphocyte overexpressed genes were down-regulated in CRPC patients with a worse prognosis. None of the genes in Fig. 5 were overexpressed in B cells. Thus, the two existing prognostic models reflect only portions of the underlying expression changes.

### A four-gene model was derived from stable co-expression modules

To comprehensively reflect all biological processes, we selected one representative gene from the functional core of each of the six stable co-expression modules to construct a prognostic model. In this study, we chose the most significantly differentiated gene between high-risk and low-risk groups in the Olmos dataset in each functional core to represent the activity of the co-expression module. Using the Olmos dataset as the training dataset and naïve Bayesian as the learning algorithm, we thus built prognostic models from the six representative genes or a subset of them. To select the optimal number of genes to include in the final model, we used leave-one-out cross-validation to assess the performance of different models (see Methods for details). We derived a four-gene model that performed best in the cross-validation tests (Additional file 1: Figure S3, estimate hazard ratio (HR) = 2.65, *P* value of log rank test = 0.004). The four genes included in our final model were *MCM2* from “up_module_1”, *PROS1* from module “up_module_2”, *CD22* from module “down_module_1”, and *TMEM66* from module “down_module_2”.

### Assessing the four-gene model in validation set I

**Fig. 6:**
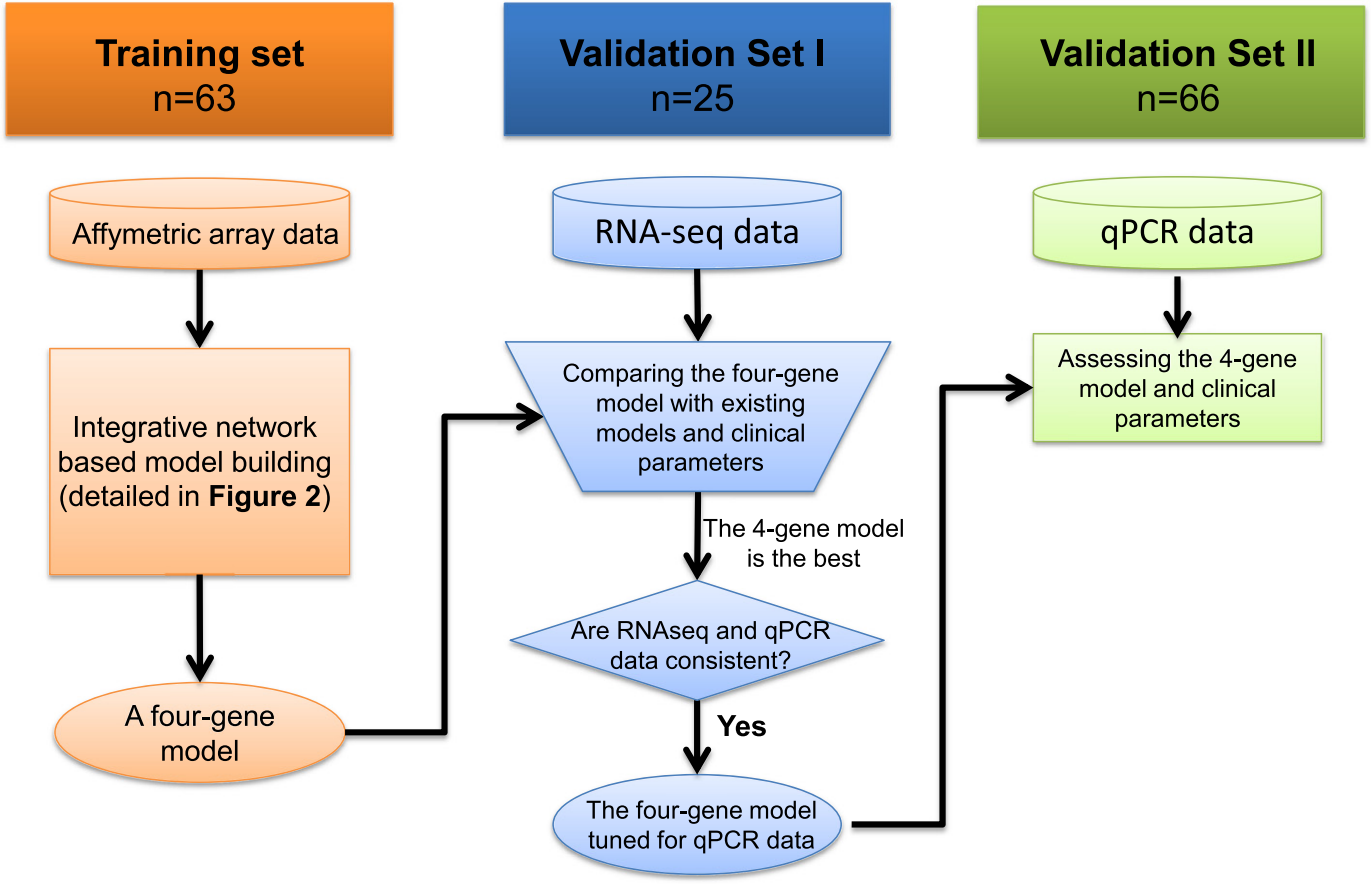
Flowchart of the multistage and multi-platform evaluation of the four-gene model

Next, we evaluated the performance of the four-gene prognostic model in two independent datasets. The evaluation procedure is outlined in Fig. 6. The first independent validation dataset consisted of 25 CRPC patients recruited at Mount Sinai Medical Center. Baseline patient characteristics are listed in Table 1. The whole blood gene expression profile for each patient was generated using RNA-seq technology. We calculated the scores of the two published whole blood gene expression-based prognostic models and the four-gene score after adjusting for platform differences (see Methods) and compared their prognostic utility in three ways.

**Table 1:** Characteristics of patients in validation sets I and II

		Validation set I (n = 25)	Validation set II (n = 66)
Age, years		71 (68, 77)	69 (63, 73)
Gleason ≤6		1 (4.8 %)	5 (10 %)
Gleason = 7		5 (24 %)	16 (32 %)
Gleason ≥8		15 (71 %)	29 (58 %)
Bone metastasis		19 (76 %)	37 (59 %)
Visceral metastasis		3 (12 %)	26 (41 %)
PSA (ng/mL)		38 (7, 437)	221 (39, 657)
Hemoglobin (g/dL)		11.9 (11.2, 13.3)	11.1 (10.0, 12.4)
LDH (U/L)		223 (200, 273)	342 (256, 561)
AP (IU/L)		84 (69, 194)	163 (85, 399)
Prior treatment	Docetaxel	7 (28 %)	52 (79 %)
	Abiraterone	4 (16 %)	11 (17 %)
	Cabazitaxel	5 (20 %)	0
	Sipuleucel-T	3 (12 %)	0
	Enzalutamide	2 (8 %)	0
Number of different prior treatments	0	13 (52 %)	14 (21 %)
	1	4 (16 %)	41 (62 %)
	2	8 (32 %)	11 (17 %)
Median follow-up, months		28.9	30.8
Number of events		15	58

Data are median (0.25 quantile, 0.75 quantile) or count (%). Median follow-up time was calculated based on survivors. All samples in validation set II were drawn right before the next treatment. The samples in validation set I were obtained either right before the next treatment or between two treatments. None of these blood samples were collected immediately after a treatment to reduce the acute impact of treatments

**Fig. 7:**
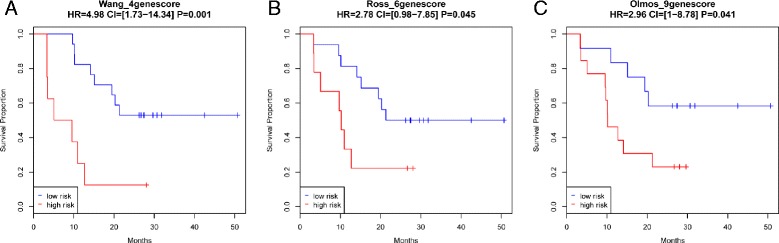
Survival curve of high- and low-risk patients in the first validation set based on Wang_4genescore (**a**), Ross_6genescore (**b**) and Olmos_9genescore (**c**) calculated using RNAseq measurement with predefined cutoffs

First, the model score was treated as a continuous value and its association with survival outcome was assessed using a univariate Cox proportional hazards model. As shown in Table 2A, all three gene models were significantly associated with survival outcome, with the four-gene score (Wang_4gene score) being the most significant. Second, we compared the independent information carried by each model score by including pairs of model scores in the bivariate Cox proportional hazard model (Table 2B). Conditioned on the four-gene score, neither the Olmos nor the Ross scores remained associated with survival (*P* = 0.4 for Olmos score and *P* >0.9 for Ross score). In contrast, the four-gene score remained significantly associated with survival when conditioning on either of the two existing model scores (*P* = 0.048 conditioning on Olmos score and *P* = 0.010 conditioning on Ross score). These comparisons suggest that the four-gene model captures information associated with survival independent from existing models. Third, a predefined cutoff was applied to the model score to partition patients into high- and low-risk groups. For the four-gene score, a universal cutoff of 0.5 was used. For the Ross six-gene score, a cutoff of 21.21 was used as suggested by the original publication. The median value was used for the Olmos nine-gene score (Additional file 1). The survival curves for low- and high-risk groups defined by each score are shown in Fig. 7. The two defined groups based on the four-gene score were most significantly different (HR = 4.98 and log rank test *P* = 0.001). In summary, all three comparisons in this validation dataset reveal that the newly derived four-gene score predicts survival better than the two previously published models.

**Table 2:** Univariate Cox regression modeling for the overall survival using each of the three gene models (A) and bivariate Cox regression modeling by combining two of the three gene models (B) in validation set I

A. Univariate analysis (individual gene model)			
	Concordance index	*P* value (Likelihood ratio test)	*P* value (Logrank test)
Wang_4genescore	0.81	0.0006	7×10^−05^
Olmos_9genescore	0.72	0.004	0.005
Ross_6genescore	0.68	0.028	0.026
B. Bivariate analysis (Combining two gene models)			
	*P* value (Wald’s test)		
Wang_4genescore	0.042		
Olmos_9genescore	0.35		
Wang_4genescore	0.010		
Ross_6genescore	0.99		
Olmos_9genescore	0.054		
Ross_6genescore	0.40

Finally, we compared the performance of the four-gene score with known clinical parameters using a univariate Cox regression analysis (Table 3A). The significant clinical parameters (*P* <0.05 in Table 3A) were then included in a multivariate analysis together with the four-gene score (Table 3B). The four-gene score was the only variate with *P* <0.05 in the multivariate analysis. It is of note that the sample size here was small for developing multivariate models. Nevertheless, the fact that the four-gene score remained significant (*P* <0.05) in multivariate analysis indicates that it carried additional predictive power independent of prognostic clinical factors.

**Table 3:** Univariate Cox regression modeling for the overall survival using each of the clinical parameters (A) and multivariate Cox regression modeling by combining four variables (*P* <0.05 in univariate analysis) (B) in validation set I. All the variables (except the metastasis site) were considered as continuous values

A. Univariate analysis of validation set I			
	Concordance index	*P* value (Likelihood ratio test)	*P* value (Logrank test)
Wang_4genescore	0.81	0.0006	7×10^−05^
Hemoglobin	0.75	0.001	0.001
VisceralMetastasis	0.58	0.006	1×10^−05^
LDH	0.72	0.043	0.016
BoneMetastasis	0.56	0.051	0.15
EosinophilCount	0.69	0.052	0.068
PSA	0.62	0.12	0.066
AP	0.61	0.18	0.13
NLRatio	0.57	0.18	0.14
MonocyteCount	0.46	0.19	0.11
PlateletCount	0.62	0.3	0.3
NeutrophilCount	0.50	0.4	0.4
Gleason	0.52	0.7	0.7
LymphocyteCount	0.59	0.7	0.7
BasophilCount	0.48	>0.9	>0.9
B. Multivariate analysis of validation set I			
	*P* value (Wald’s test)		
Wang_4genescore	0.045		
Hemoglobin	0.18		
visceralMets	0.13		
LDH	0.4		

### Validating expression levels of genes used in the four-gene model by qPCR

**Fig. 8:**
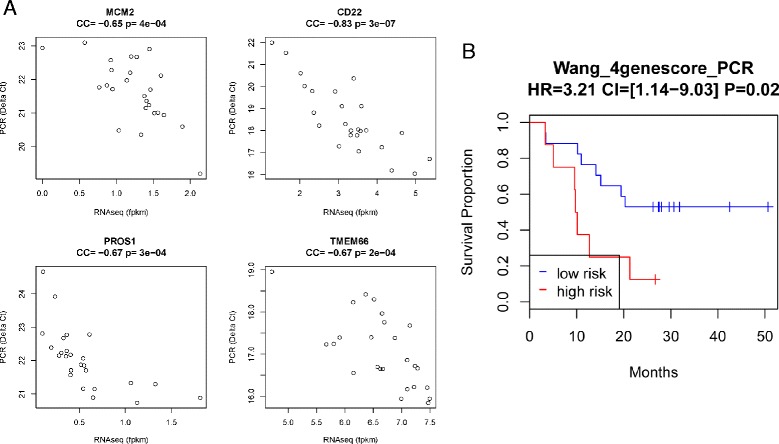
**a** Correlation between PCR and RNAseq measurements of the four-gene expression. **b** Survival curve of high- and low-risk patients in the first validation set based on Wang_4genescore calculated using PCR measurement

Before further assessing the four-gene prognostic model in additional validation sets, we measured the gene expression levels of the four genes using the same blood samples collected from 25 CRPC patients in the validation set I on the ViiA7 qPCR instrument using custom-made Taqman Array Cards. The correlations between the RNAseq and PCR measurements for the four genes were within an appropriate range (Fig. 8a, Pearson’s correlation coefficient >0.6). The four-gene score calculated using qPCR measurements was also able to partition patients into low- and high-risk groups with significantly different survival times (HR = 3.21, log rank test *P* = 0.02; Fig. 8b). Thus, the four-gene model developed in the Olmos dataset (profiled using Affymetrix arrays) was validated in an independent dataset, validation set I, using both RNAseq and qPCR after linear transformation to adjust for platform differences.

### Optimizing the four-gene model based on qPCR

Since the qPCR platform is more cost efficient than RNAseq in practical applications, we used it to further validate the four-gene model. We fine-tuned the parameters of the four-gene model based on qPCR measurements in validation set I so that there was no need to correct for platform differences each time. In particular, we selected nine high-risk patients (survival time <12 months) and 10 low-risk patients (survival time >24 months) from validation set I. We then trained a linear model of the four genes to distinguish the two patient groups using logistic regression. The resulting linear formula was s = −27.28–3.43×MCM2–0.68×PROS1+3.06×CD22+3.49×TMEM66, and Wang_4genescore was calculated as exp(s)/(exp(s)+1). The linear model was trained based on the qPCR measurement of the four genes (gene expression in the formula refers to the delta T measurement in qPCR) and the coefficients in the formula were specifically optimized for the qPCR platform.

### Evaluating the four-gene model in validation set II

**Fig. 9:**
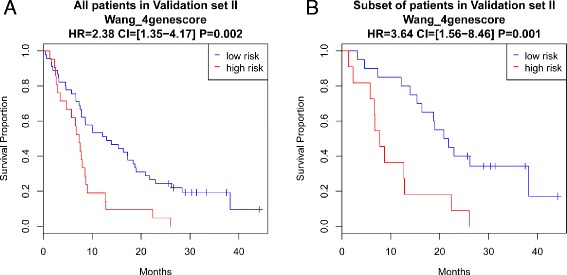
Survival curve of high- and low-risk group in the second validation set based on Wang_4genescore when all patients are considered (**a**) and when patients with visceral metastasis or under the third line treatment are removed (**b**)

The second independent dataset, validation set II, consisted of 66 CRPC patients recruited from the Urology Clinic at the University of Technology in Munich, Germany. Patient characteristics are listed in Table 1. Expression levels of the four genes were measured using qRT-PCR and the four-gene scores were calculated using the formula noted. Patients were partitioned into high- and low-risk groups according to the four-gene score using a universal cutoff of 0.5. The two groups had significantly different survival outcomes (*P* = 0.002, Fig. 9a). It is worth noting that, although not statistically significant, the estimated HR (HR = 2.38) was smaller than in validation set I (HR = 3.21 and 4.98 for qPCR and RNAseq measurements, respectively). The lower HR or prognostic power in validation set II was likely caused by patient characteristic differences in the two datasets: validation set II included many more advanced patients and patients with heavier prior treatments. For instance, 41 % of the patients in validation set II had visceral metastasis, while only 12 % in the first set did. In addition, 79 % of the patients in validation set II had received prior treatment compared to 48 % in the first set. We noted survival curves were different between visceral metastasis and no visceral metastasis and between patients receiving first, second, and third line treatment (Additional file 1: Figure S4). As a result, the risk of death by 24 months was much higher in validation set II (87 %) as compared to validation set I (60 %). If patients with visceral metastasis or having third line treatment were removed from the analysis, the estimated HR of the four-gene score increased (HR = 3.64; Fig. 9b). On the other hand, the estimated HR decreased if only patients with visceral metastasis or having third line treatment were considered (HR = 2.14; Additional file 1: Figure S5). Thus, a future multivariate analysis combining these clinical parameters and the four-gene score is warranted in a larger cohort.

Univariate and multivariate analysis of clinical parameters was conducted in this group and again suggested that the four-gene score carried additional prognostic power independent of clinical parameters (Table 4). In addition, multivariate analysis revealed that the presence of visceral metastasis and treatment line was complementary to the four-gene score. Thus, the analysis suggests that combining certain clinical parameters with the four-gene score could provide better performance in predicting overall survival.

**Table 4:** Univariate Cox regression modeling for the overall survival using each of the clinical parameters (A) and multivariate Cox regression modeling by combining seven variables (*P* <0.05 in univariate analysis) (B) in validation set II. All the variables (except the metastasis site and treatment line) were considered as continuous values

A. Univariate analysis of validation set II			
	Concordance index	*P* value (Likelihood ratio test)	*P* value (Logrank test)
Hemoglobin	0.65	7×10^−05^	6×10^−05^
LDH	0.68	0.0004	7×10^−06^
Wang_4genescore	0.60	0.002	0.0007
ThirdLineTreatment	0.56	0.006	0.002
AP	0.63	0.008	0.001
VisceralMetastasis	0.58	0.043	0.038
LeucocyteNr	0.60	0.048	0.045
NLR	0.56	0.15	0.095
Lymphocytes_percent	0.58	0.3	0.3
SecondLineTreatment	0.55	0.3	0.3
FirstLineTreatment	0.51	0.4	0.4
BoneMetastasis	0.51	0.4	0.4
Gleason	0.53	0.6	0.6
Neutrophile_percent	0.47	>0.9	>0.9
PSA	0.57	>0.9	>0.9
B. Multivariate analysis of validation set II			
	*P* value (Wald’s test)		
Hemoglobin	0.2		
LDH	0.3		
Wang_4genescore	0.03		
ThirdLineTreatment	0.002		
AP	0.9		
visceralMets	0.05		
LeucocyteNr	0.07		

## Discussion

Herein, we developed a module-based integrative computational strategy to construct robust prognostic models from expression profiles by dissecting candidate genes into stable co-expression modules that were functionally related to cancer progression. The advantages of our strategy and the resulting four-gene model are summarized below.

First, in selecting signature genes to be included in the model, we focused on stable co-expression modules that reflect the activity of biological pathways rather than individual genes. It is not a ‘black box’ learning approach, but rather a gene-selection approach guided by underlying biology. We showed that all of the up-regulated modules were overexpressed in myeloid cells and all of the down-regulated modules were over-expressed in lymphoid cells. A simplistic interpretation would be that observed mRNA expression changes may represent alterations in the composition of hematopoietic cells during prostate cancer progression. However, the four-gene score performed better than cell count-based clinical parameters in both validation datasets (Tables 3 and 4), suggesting that cell component change was only one factor contributing to the patients’ prognosis. For example, there was a significant correlation between the gene expression level of TMEM66 (overexpressed in T cells) and lymphocyte count (Additional file 1: Figure S6A, Pearson’s correlation coefficient = 0.48), indicating TMEM66 expression level reflected lymphocyte cell abundance change. However, TMEM66 gene expression level predicted patient survival much better than lymphocyte cell count using a bivariate cox regression model (*P* = 0.002 and 0.2 for TMEM66 and lymphocyte count, respectively), suggesting TMEM66 gene expression level carried more prognostic information than T cell or change in lymphocyte counts. Another related cell count-based clinical measurement is the neutrophil to lymphocyte ratio (NLR), which has been shown to be prognostic in several cancer studies [28–31]. We similarly observed a trend of patients with higher NLR having a worse survival outcome (Additional file 1: Figure S7). However, since the HR was relatively small (1.52 and 1.38 for validation sets I and II) and the sample size in our study was smaller than those of the previous studies, the prognostic power of NLR was not statically significant in our validation sets (Tables 3 and 4, *P* >0.05). While there was a significant correlation between the four-gene score and the NLR in our study (Additional file 1: Figure S6B, Pearson’s correlation coefficient = 0.55), our four-gene score demonstrated much better prognostic power than NLR. We reason that beside cell count changes, gene expression levels also reflect cellular or pathway activity, and it is likely that the alteration of both the abundance and activity of different cells eventually leads to differential prognostic outcomes. Another explanation is that the expression change also reflects a combination of cell count changes of multiple types or sub-types of cells which were not directly measured in our study. The observation that up-regulated stable co-expression modules were also overexpressed in early erythroid cells, myeloid progenitor cells, and hematopoietic stem cells suggests that their up-regulation may come from myeloid-derived cells whose counts are not routinely measured. For example, they may represent myeloid progenitor cells which have ‘leaked’ from bone marrow due to metastasis [32] or circulating myeloid-derived suppressor cells, which have been shown to greatly influence tumor progression and metastasis [33].

Second, the module-based procedure enabled us not only to comprehensively represent diverse pathways but also to distinguish biological signals from data-specific ‘noise’. There are many advanced machine learning algorithms (e.g. Lasso [34] and ElasticNet [13]) which can automatically select the best set of features to be included in the model. However, since the features are usually learned entirely from the training dataset, they may be biased to dataset-specific effects. For instance, the model trained using ElasticNet showed high accuracy in the training dataset by cross-validation, but such high accuracy failed to be reproduced in the independent validation datasets (Additional file 1: Figures S8 and S9 and Supplementary Methods in Additional file 1).

Third, the new four-gene model was evaluated in a multi-stage, multi-platform, and multi-institutional process. The training dataset and the two validation datasets were generated from CRPC cohorts recruited at three different institutions using three different platforms, i.e. Affymetrix array, RNAseq, and qPCR. Our four-gene model performed extremely well across all of these datasets with a universal cutoff value. We also showed that the four-gene score was stable for intra-patient and inter-day blood samples and the four-gene score changed along with disease progression. More details about the four-gene score variability can be found in Additional file 1.

There are many important clinical and translational implications to these data. First, if host immune function is so reproducibly critical to prostate cancer progression and survival, then current efforts to model therapeutic efficacy in certain models, such as patient-derived xenografts, will likely fail to represent the true outcome in patients. Second, the current development of promising immunotherapies in cancer, including vaccines, checkpoint inhibitors, and other immunomodulatory agents, will clearly need improved biomarkers to predict benefit and to better guide personalized therapies. Whole blood RNA profiles hold great promise in evaluating such baseline and serial changes in immune parameters, given its ability to provide a potentially holistic view of the key RNA transcripts involved in clinical benefit. Finally, clinical trial stratification using prognostic and predictive models based on whole blood RNA profiles will enable more rapid drug development by targeting specific populations with differential outcomes in CRPC but also with different baseline characteristics that would be more likely to benefit from specific therapies.

Despite these encouraging findings, there are important limitations and unaddressed questions that need further study. For instance, some alternative biomarker approaches, such as circulating tumor cell count [35], were not directly compared in this study. Halabi et al. [36, 37] described how standard clinical variables can be used to predict prognosis for CRPC. While we included as many clinical parameters available to us, there were several variables not available in our current study (e.g. opioid analgesic use and Eastern Cooperative Oncology Group performance status). Follow-up studies are needed to uncover the causal and mechanistic interactions between blood gene expression changes and clinical disease progression.

## Conclusions

In summary, we developed a four-gene model which provides a robust and minimally invasive approach for determining prognosis of CRPC patients using peripheral blood gene expression. The initial results are promising and the next step would be validation in a cohort of suitable size to quantify the prognostic improvement by the gene score upon the standard set of clinical parameters. The novel module-based computational strategy described herein may have broader applications, and significant impact, in precision medicine.

## Additional file

Additional file 1:
**This file includes the supplementary methods, Tables S1–S3 and Figures S1–S9.** (DOCX 2139 kb)

## Abbreviations

CRPC: Castration-resistant prostate cancer
FPKM: Fragments per kilobase of exon per million fragments mapped
GTEx: Genotype-Tissue Expression study
HR: Hazard ratio
IFB: Iceland Family Blood study
NLR: Neutrophil to lymphocyte ratio
