# Supplementary material for: A robust blood gene expression-based prognostic model for castration-resistant prostate cancer
Source: BMC Med. 2015 Aug 21;13:201. doi: 10.1186/s12916-015-0442-0 (PMC4546313; doi:10.1186/s12916-015-0442-0)
Supplement: Additional file 1: — This file includes the supplementary methods, Tables S1–S3 and Figures S1–S9. (DOCX 2139 kb) [file 12916_2015_442_MOESM1_ESM.docx]

**Supplementary Materials for**

**A robust blood gene expression-based prognostic model for castration-resistant prostate cancer**

Li Wang^1,2$^, Yixuan Gong^3$^, Uma Chappada-Venkata^3^, Matthias Heck^4^, Margitta Retz^4^,

Roman Nawroth^4^, Matthew Galsky^3^, Che-Kai Tsao^3^, Eric Schadt^1,2,3^, Johann De Bono^5^, David Olmos^6^,

Jun Zhu^1,2,3,*^, William K. Oh^3,*^

^1^Icahn Institute for Genomics and Multiscale Biology; ^2^Department of Genetics and Genomic Sciences; ^3^The Tisch Cancer Institute, Icahn School of Medicine at Mount Sinai, NY, USA; ^4^Urological Clinic, University of Technology, Munich, Germany; ^5^Institute for Cancer Research, Royal Marsden Hospital, ^6^Spanish National Cancer Research Centre.

^$^Contributed equally

*Addresses for correspondence:

Dr. Jun Zhu

Icahn Institute for Genomics and Multiscale Biology

Icahn School of Medicine at Mount Sinai

New York, NY 10029

[jun.zhu@mssm.edu](mailto:jun.zhu@mssm.edu)

or

Dr. William K. Oh

The Tisch Cancer Institute

Icahn School of Medicine at Mount Sinai

New York, NY 10029

William.oh@mssm.edu

Supplementary Methods

### Model training and cross-validation

In each round of cross-validation, we assigned one sample in the Olmos dataset as the testing sample and the rest as the training dataset. From the training dataset, we repeated from step 1) to step 4) and generated two ranked lists of representative genes, each containing *n* genes (*n=3* in this study). We then built *n* naïve Bayesian classifiers by including different number of top representative genes, i.e., the *i*th model includes the top *i* representative genes from each ranked list, *i* from 1 to *n*. We then predicted the testing sample to be in the low risk or high risk group using each classifier (the posterior probability cutoff = 0.5). We repeated the round of cross-validation with a different sample as testing sample each time until all samples had been tested. We then compared the KM survival curves of the low risk and high risk group predicted by models with different number of genes in the cross-validation, and identified the best performing model by which the two groups showed the most significantly different survival outcomes (p-value of log rank test). The final model was built based on all samples in the Olmos dataset and using the same number of top ranking representative genes in the best performing model in cross-validation.

**The four-gene model performed better than the gene model generated using ElasticNet**

We generated a gene model by the widely used machine learning algorithm ElasticNet and compared it with our four-gene model. Similar to the procedure for the four-gene model, we used the Olmos dataset as a training dataset, and leave-one-out cross-validation to tune the parameters for the ElasticNet algorithm. The cross-validation result of the ElasticNet algorithm was better than the four-gene score (Figure S5, p-value of log rank test = 9.3e-05). The final model generated by ElasticNet consisted of fifteen genes (ElasticNet_15genesocre), whose expression profiles across different types of hematopoietic cells were shown in Figure S6. Unlike Olmos_9genescore, genes in ElasticNet_15genescore were overexpressed in many different cell types. This is consistent with the nature of the ElasticNet algorithm, which can automatically select a few genes among many highly correlated genes and thus could potentially represent many distinct underlying pathways. However, when we applied the ElasticNet_15genescore to the independent validation dataset, there was no significant or even marginally significant prognostic power (p-value of likelihood ratio test in the univariate Cox proportional hazard model =0.62 when the gene score was treated as continuous variable, and p-value of log rank test=0.36 when treated as discrete variable with cutoff of 0.5). Thus the high accuracy in the cross-validation represents an overfitting to the Olmos dataset. Such an overfitting is not uncommon given the incapacity of ElasticNet algorithm to distinguish stable correlations from random or dataset-specific ones. In fact, none of the fifteen genes are within the stable co-expression modules. In summary, the model generated by ElasticNet showed high accuracy in the training dataset by cross-validation, but such high accuracy failed to be reproduced in the independent validation dataset.

**Intra-patient and Inter-day variability of 4-genescore**

To assess the stability of 4-genescore within short period of time, we measured the gene expression of two consecutive blood draws (<3 months) from four patient using qPCR. As shown in Figure S10A, the calculated 4-genescore remains relatively stable for each patient within this short period of time. When the cutoff of 0.5 was applied to categorize patients into high-risk and low-risk group, each patient was categorized into the same risk group based on the 4-gene scores derived from the two consecutive blood draws. This result suggests of the results of the assay for each patient within short period of time frame are stable. To compare short term variation with dynamic changes of the 4-genescore in longer time period, we obtained time series data for three patients ranging 20-30 months using RNAseq. The 4-genescore showed a clear up-ward trend for all three patients, likely reflecting the underlying disease progression (Figure S10B). The closer the blood draw time was to the deceased time or the censored time, the higher risk the 4-gene score predicted the patient with. Thus, our assay and the resulting 4-genescore not only present relative reproducible prognosis prediction, but also can be potentially used to monitor disease progression.

Supplementary Tables and Figures

Table S1 Enriched gene sets for stable co-expression modules. The gene set information was obtained from the curated gene sets in MsigDB database. The third column is the overlapping gene count between that module and the corresponding gene set. The forth column is the p-value of fisher’s exact test for enrichment.

| **Module Name** | **Gene Set Name** | **Count** | **P-value** |
| --- | --- | --- | --- |
| Up_module_1 | REACTOME_CELL_CYCLE_MITOTIC | 28 | 3.30E-28 |
| Up_module_1 | REACTOME_CELL_CYCLE | 29 | 8.50E-27 |
| Up_module_1 | KEGG_CELL_CYCLE | 15 | 3.10E-16 |
| Up_module_1 | REACTOME_DNA_REPLICATION | 15 | 1.10E-13 |
| Up_module_1 | PID_PLK1_PATHWAY | 9 | 4.30E-12 |
| Up_module_1 | REACTOME_MITOTIC_M_M_G1_PHASES | 13 | 9.10E-12 |
| Up_module_1 | REACTOME_MITOTIC_PROMETAPHASE | 10 | 5.50E-11 |
| Up_module_1 | PID_AURORA_B_PATHWAY | 8 | 1.30E-10 |
| Up_module_1 | REACTOME_CYCLIN_A_B1_ASSOCIATED_EVENTS_DURING_G2_M_TRANSITION | 6 | 2.10E-10 |
| Up_module_1 | KEGG_OOCYTE_MEIOSIS | 10 | 9.80E-10 |
| Up_module_1 | REACTOME_MITOTIC_G1_G1_S_PHASES | 10 | 3.20E-09 |
| Up_module_1 | PID_FOXM1PATHWAY | 7 | 3.90E-09 |
| Up_module_1 | REACTOME_KINESINS | 6 | 5.10E-09 |
| Up_module_1 | PID_E2F_PATHWAY | 8 | 8.50E-09 |
| Up_module_1 | REACTOME_G1_S_TRANSITION | 9 | 1.00E-08 |
| Up_module_1 | REACTOME_MITOTIC_G2_G2_M_PHASES | 8 | 2.00E-08 |
| Up_module_1 | REACTOME_CELL_CYCLE_CHECKPOINTS | 8 | 2.80E-07 |
| Up_module_1 | REACTOME_E2F_MEDIATED_REGULATION_OF_DNA_REPLICATION | 5 | 3.70E-07 |
| Up_module_1 | REACTOME_REGULATION_OF_MITOTIC_CELL_CYCLE | 7 | 5.10E-07 |
| Up_module_1 | KEGG_PROGESTERONE_MEDIATED_OOCYTE_MATURATION | 7 | 7.40E-07 |
| Up_module_1 | REACTOME_G1_S_SPECIFIC_TRANSCRIPTION | 4 | 1.30E-06 |
| Up_module_1 | REACTOME_G2_M_CHECKPOINTS | 5 | 1.80E-06 |
| Up_module_1 | PID_ATR_PATHWAY | 5 | 4.50E-06 |
| Up_module_1 | REACTOME_G0_AND_EARLY_G1 | 4 | 7.00E-06 |
| Up_module_1 | REACTOME_MHC_CLASS_II_ANTIGEN_PRESENTATION | 6 | 8.90E-06 |
| Up_module_1 | BIOCARTA_RANMS_PATHWAY | 3 | 3.50E-05 |
| Up_module_1 | PID_AURORA_A_PATHWAY | 4 | 3.50E-05 |
| Up_module_1 | REACTOME_S_PHASE | 6 | 3.60E-05 |
| Up_module_1 | REACTOME_FACTORS_INVOLVED_IN_MEGAKARYOCYTE_DEVELOPMENT_AND_PLATELET_PRODUCTION | 6 | 4.90E-05 |
| Up_module_1 | REACTOME_APC_C_CDH1_MEDIATED_DEGRADATION_OF_CDC20_AND_OTHER_APC_C_CDH1_TARGETED_PROTEINS_IN_LATE_MITOSIS_EARLY_G1 | 5 | 5.90E-05 |
| Up_module_1 | PID_P73PATHWAY | 5 | 9.80E-05 |
| Up_module_2 | REACTOME_RESPONSE_TO_ELEVATED_PLATELET_CYTOSOLIC_CA2_ | 5 | 7.60E-06 |
| Up_module_2 | REACTOME_PLATELET_ACTIVATION_SIGNALING_AND_AGGREGATION | 6 | 4.40E-05 |
| Up_module_2 | REACTOME_HEMOSTASIS | 8 | 6.70E-05 |
| Down_module_1 | KEGG_B_CELL_RECEPTOR_SIGNALING_PATHWAY | 7 | 1.00E-08 |
| Down_module_1 | REACTOME_ANTIGEN_ACTIVATES_B_CELL_RECEPTOR_LEADING_TO_GENERATION_OF_SECOND_MESSENGERS | 5 | 9.00E-08 |
| Down_module_1 | PID_BCR_5PATHWAY | 6 | 2.00E-07 |
| Down_module_1 | KEGG_PRIMARY_IMMUNODEFICIENCY | 5 | 2.50E-07 |
| Down_module_1 | REACTOME_SIGNALING_BY_THE_B_CELL_RECEPTOR_BCR | 6 | 4.90E-06 |
| Down_module_3 | PID_CD8TCRPATHWAY | 4 | 1.30E-05 |
| Down_module_3 | REACTOME_GENERATION_OF_SECOND_MESSENGER_MOLECULES | 3 | 2.70E-05 |
| Down_module_3 | PID_TCR_PATHWAY | 4 | 3.20E-05 |

**Table S2** Enriched gene sets of cell type specific overexpression for stable co-expression modules. Count: the overlapping gene count between that module and the corresponding gene set. Enrichment Score: the ratio of the observed overlapping gene count VS the expected overlapping gene count when the two gene sets are independent. P-value: p-value of fisher’s exact test for enrichment. ERY: Erythroid cell. GM: Granulocyte/monocyte.

| **Module Name** | **Gene Set of Cell Type Specific Overexpression** | **Count** | **Enrichment Score** | **P-value** |
| --- | --- | --- | --- | --- |
| Up_module_1 | ERY_up | 44 | 6.4 | 1.20E-33 |
| Up_module_2 | ERY_up | 15 | 3.8 | 9.20E-07 |
| Up_module_3 | GM_up | 15 | 8.5 | 3.00E-12 |
| Down_module_1 | BCELL_up | 32 | 6.6 | 8.20E-25 |
| Down_module_2 | TCELL_up | 19 | 2.4 | 6.10E-05 |
| Down_module_3 | TCELL_up | 25 | 3.0 | 5.70E-09 |

**Table S3** Enriched gene sets of cell type specific overexpression for the up-regulated and down-regulated gene list. Refer to Table S2 for more legends.

| **Module Name** | **Gene Set of Cell Type Specific Overexpression** | **Count** | **Enrichment Score** | **P-value** |
| --- | --- | --- | --- | --- |
| up-regulated genes | ERY_up | 561 | 4.8 | 0.00E+00 |
| down-regulated genes | TCELL_up | 195 | 2.0 | 1.80E-27 |
| down-regulated genes | BCELL_up | 153 | 2.4 | 1.90E-27 |

1. B)


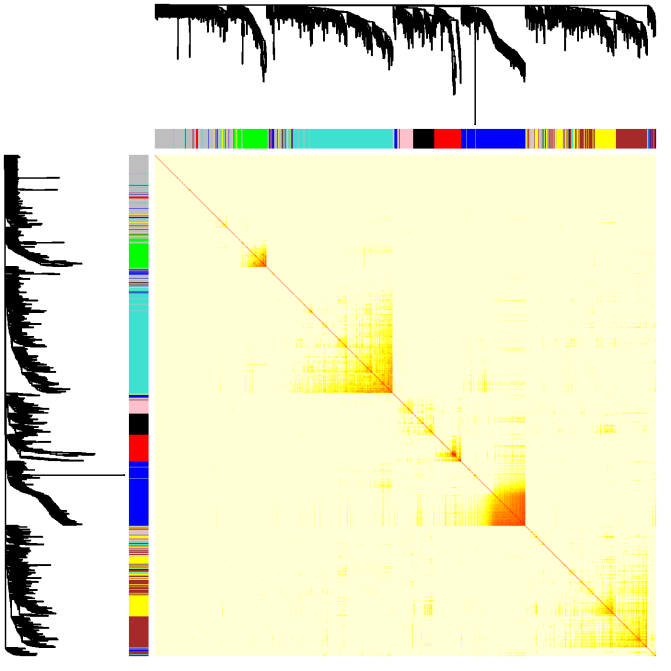

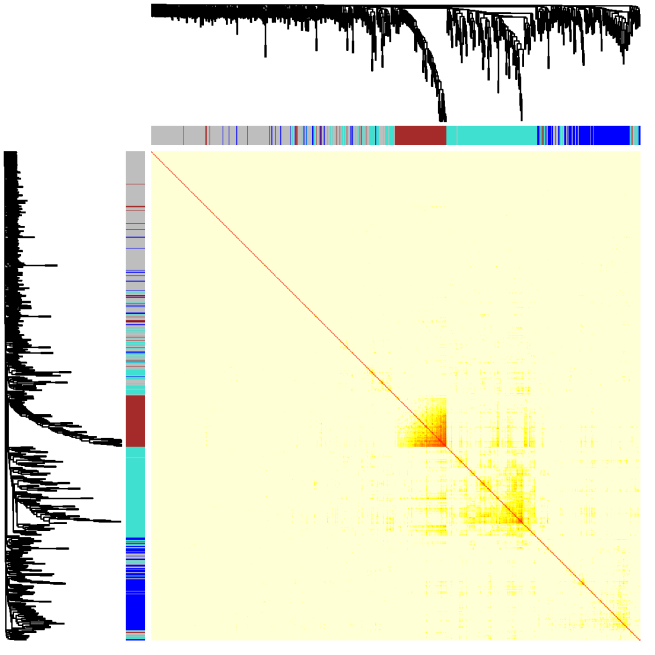


**Figure S1** Co-expression networks among genes up-regulated in high-risk CRPC patients (A) and genes down-regulated in high-risk CRPC patients (B) are constructed from whole blood mRNA profiling of 99 male samples in GTEX dataset. Light color represents low overlap and progressively darker red color represents higher overlap. The gene dendrogram and module assignment are shown along the left side and the top. Each color represents one module, and grey color represents genes that are not assigned to any modules.


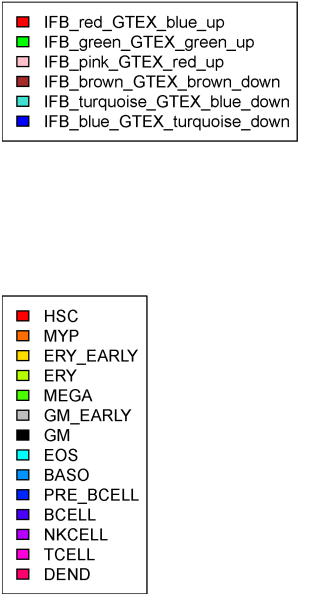

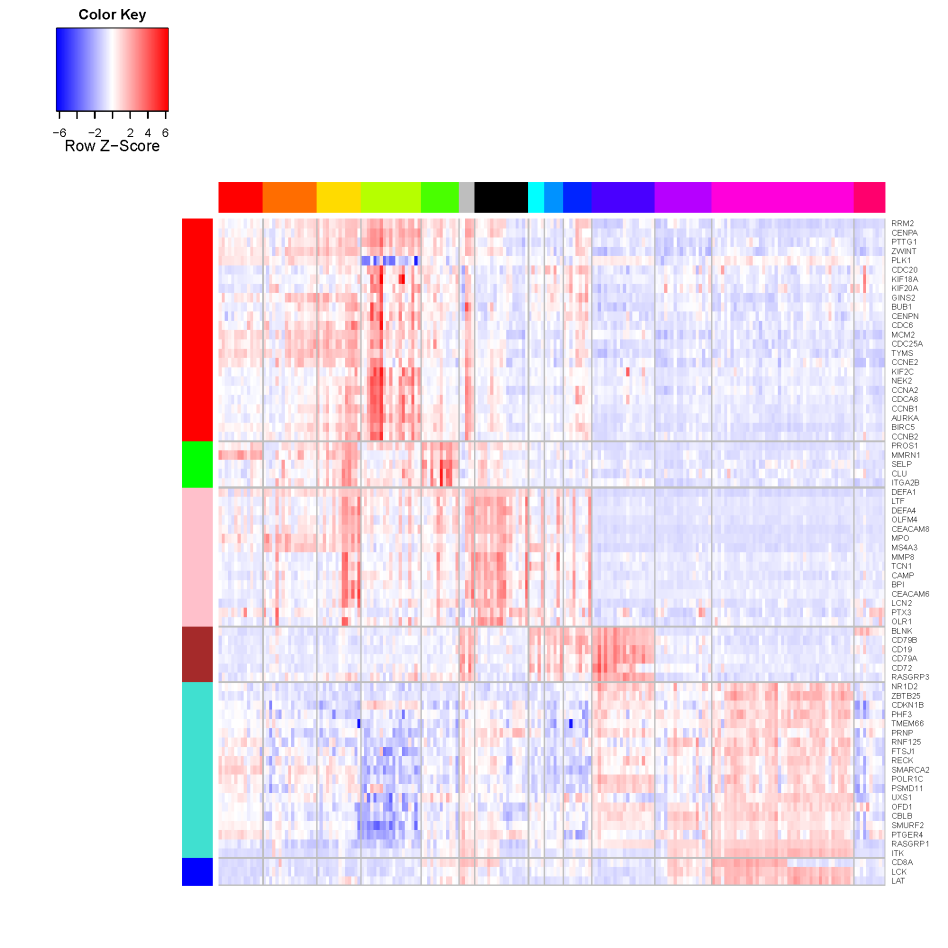


Row Legend:

Column Legend:

Figure S2 Heatmap of gene expression across different types of blood cell lines for functional cores of stable co-expression modules. Rows represent genes which are within functional cores of stable co-expression modules (row legend). Columns represent blood cell lines which are grouped according to the lineage (column legend). Some abridgements: HSC: Hematopoietic stem cell. MYP: myeloid progenitor. ERY: Erythroid cell. MEGA: megakaryocyte. GM: Granulocyte/monocyte. EOS: eosinophil, BASO: basophil. DEND: dendritic cell.

1. B) C)


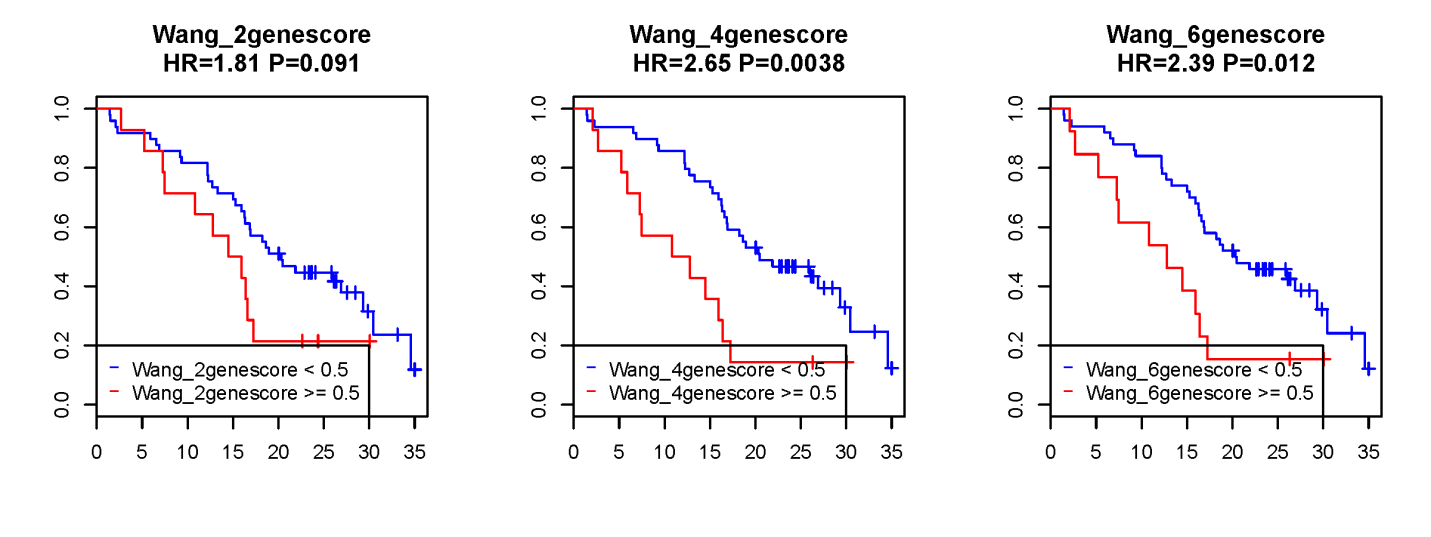


Survival Proportion

Survival Proportion

Months

Survival Proportion

Months

Months

Figure S3 Survival curves of high and low risk patients in Olmos dataset as predicted by Naïve Bayesian classifiers in leave-one-out cross validation. The Naïve Bayesian classifiers were built using top 2 (A), 4 (B) or 6 (C) representative genes selected in our module-based procedures, respectively.


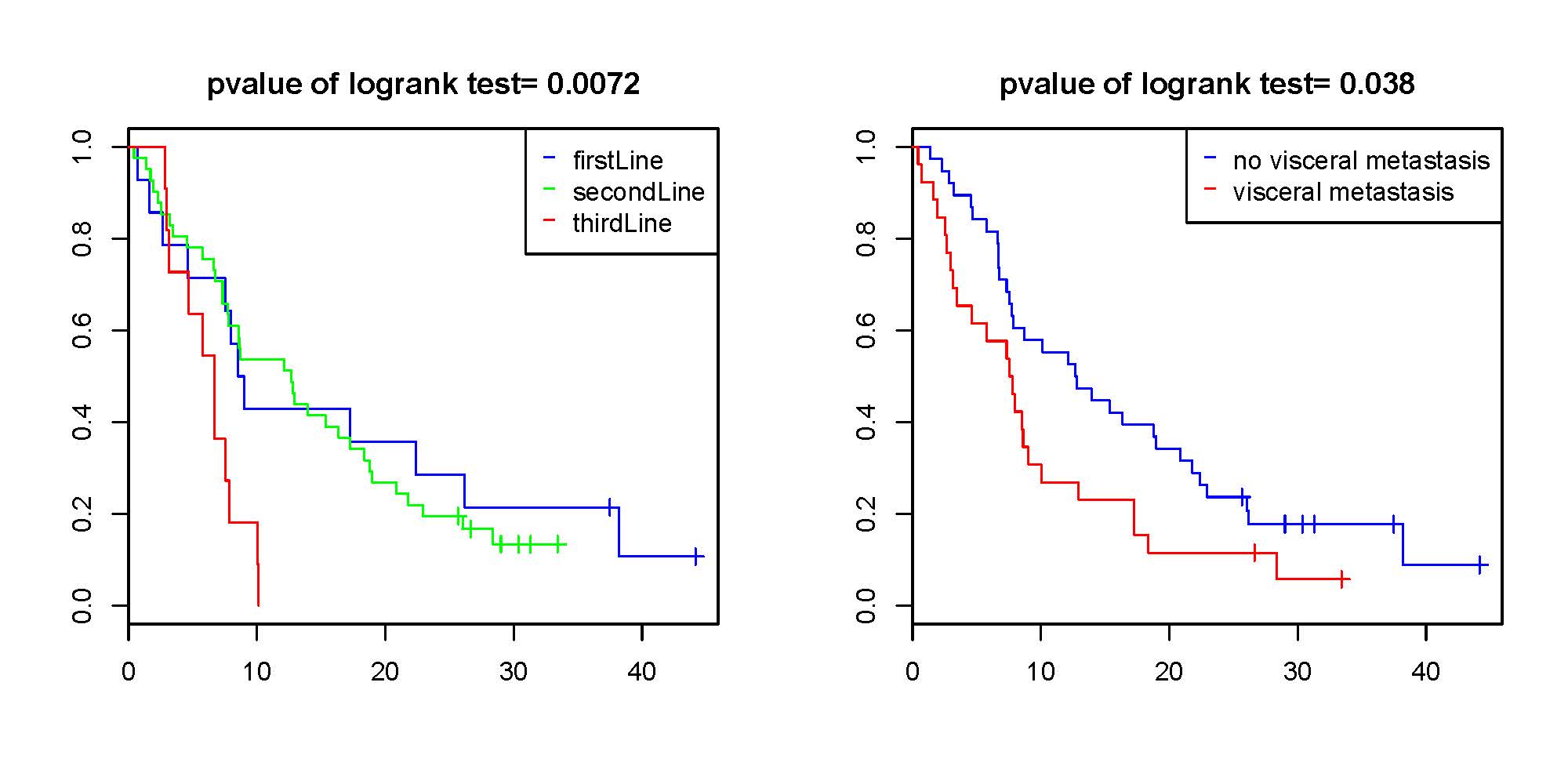


Survival Proportion

Survival Proportion

Months

Months

Figure S4 KM survival curves for patients under different treatment groups in the second independent cohort.


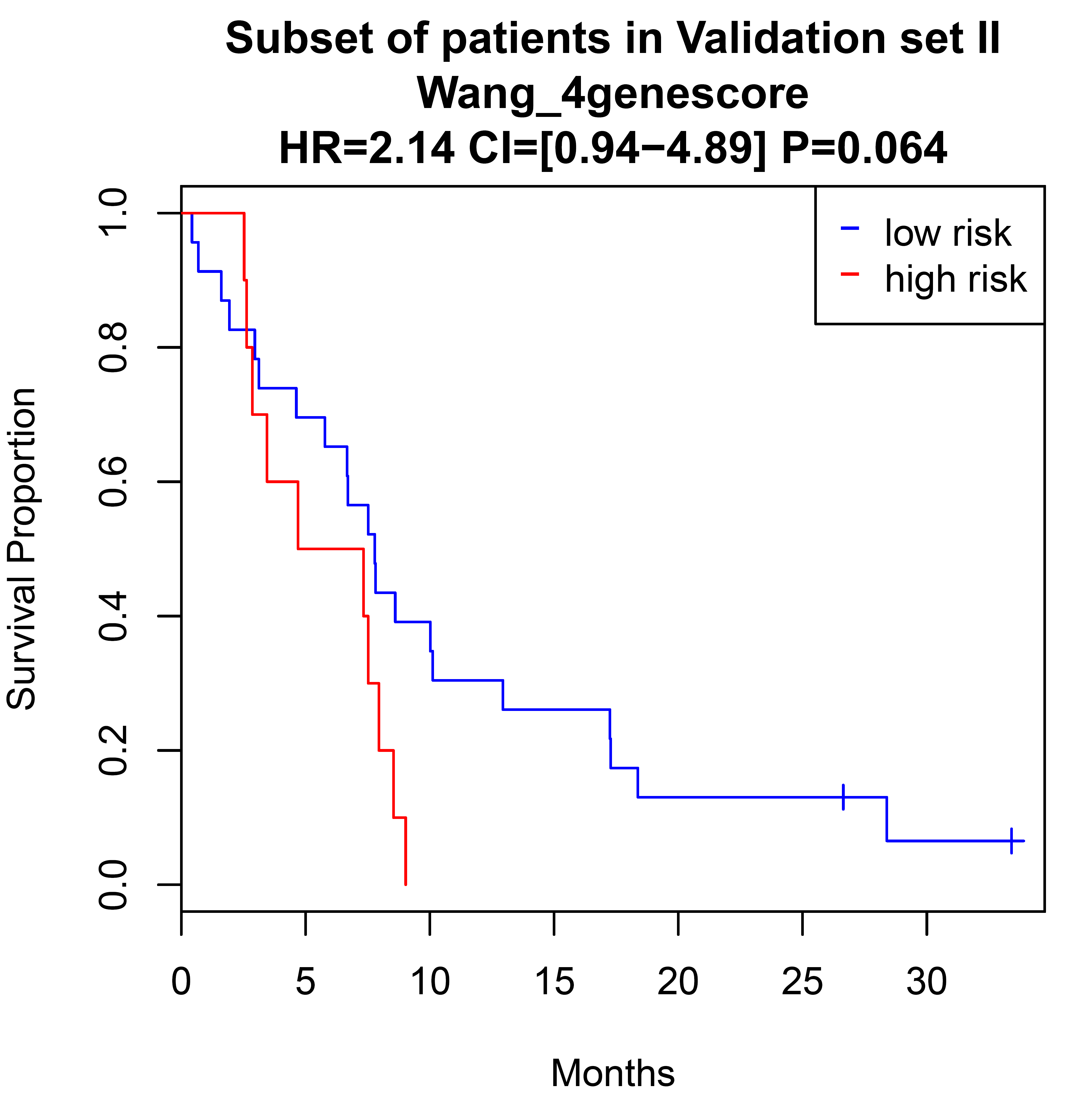


Figure S5 Survival curve of high and low risk group in the second validation set based on Wang_4genescore when only patients with visceral metastasis or under the third line treatment are considered.

A) B)

Figure S6 A) Correlation between TMEM66 gene expression and Lymphocyte count in Validation Set I. B) Correlation between NLRatio and four-gene score in Validation Set I.

 A) B)

Survival Proportion

Survival Proportion

Months

Months

Figure S7 Survival curve for patients with high and low NLRatio in Validation Set I A) and Validation Set II B).


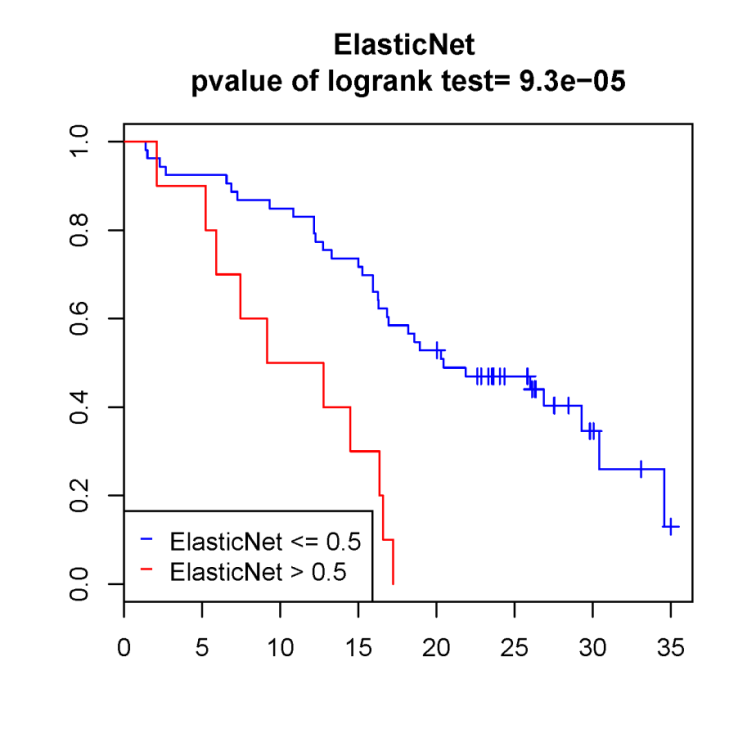


Months

Survival Proportion

Figure S8 Survival curve of high and low risk patients in Olmos dataset as predicted by ElasticNet algorithm in leave-one-out cross validation.


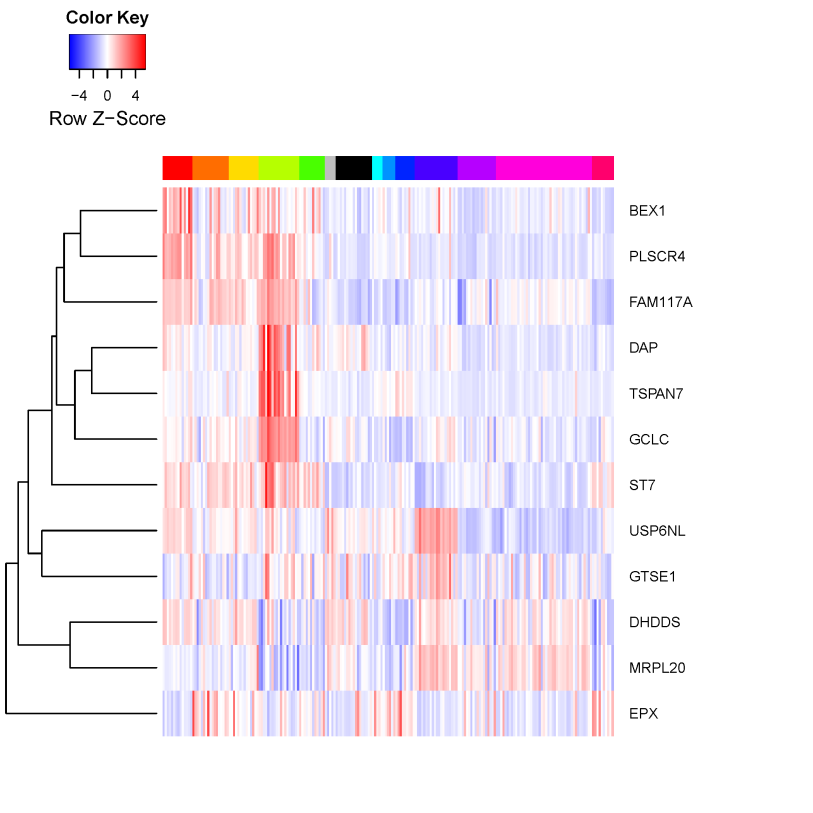

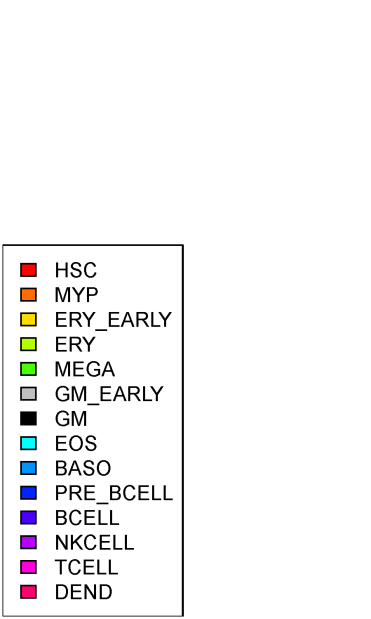


Column Legend:

Figure S9 Heatmap of gene expression across different blood cell lines for genes in various prognostic models. Rows are genes from different prognostic models (row legend), and columns are cell lines of different lineages (column legend, same as in Figure 4). Only genes with available cell line expression profiles are shown here.


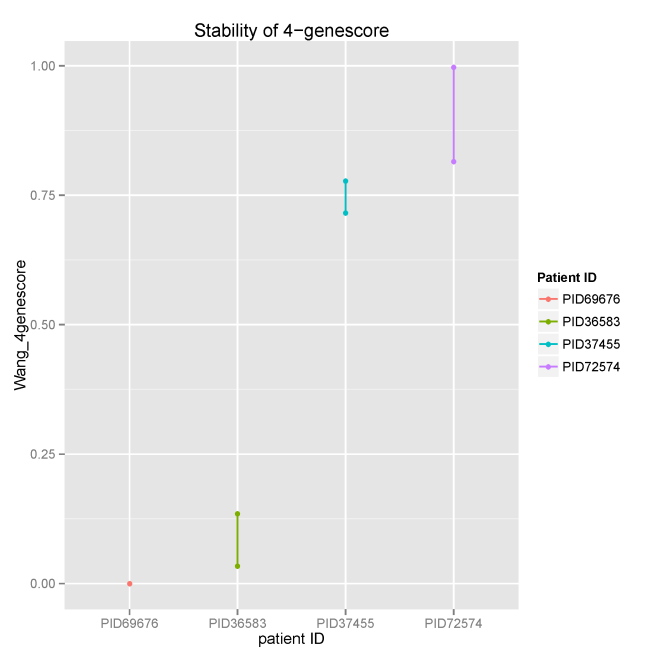

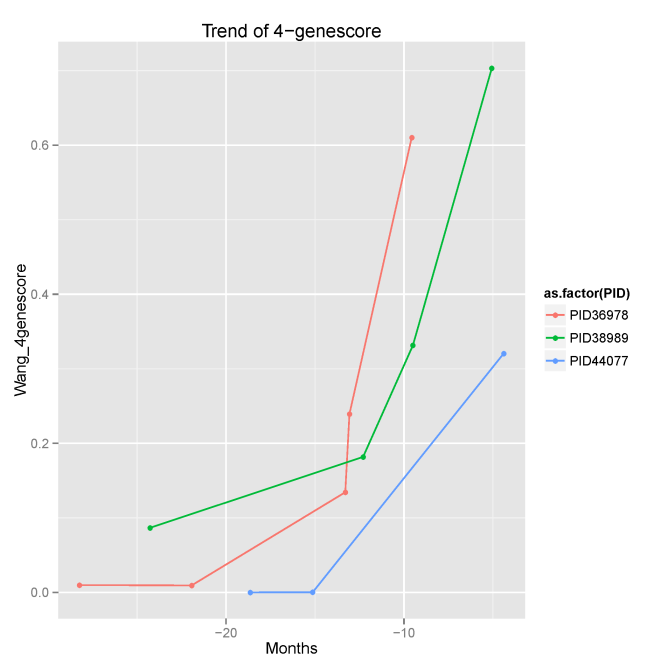
A) B)

Figure S10 A) Pairs of 4-genescore measured for the same patient within 3 months. The gene score was calculated based on qPCR readout. B) Trend of 4-genescore for each patient as disease progresses. Time zero represents the decreased time or censored time. The gene score was calculated based on RNAseq readout.
